# RMDisease V2.0: an updated database of genetic variants that affect RNA modifications with disease and trait implication

**DOI:** 10.1093/nar/gkac750

**Published:** 2022-09-05

**Authors:** Bowen Song, Xuan Wang, Zhanmin Liang, Jiongming Ma, Daiyun Huang, Yue Wang, João Pedro de Magalhães, Daniel J Rigden, Jia Meng, Gang Liu, Kunqi Chen, Zhen Wei

**Affiliations:** Department of Biological Sciences, Xi’an Jiaotong-Liverpool University, Suzhou, 215123, China; Department of Mathematical Sciences, Xi’an Jiaotong-Liverpool University, Suzhou, 215123, China; Institute of Systems, Molecular and Integrative Biology, University of Liverpool, Liverpool L7 8TX, UK; Department of Biological Sciences, Xi’an Jiaotong-Liverpool University, Suzhou, 215123, China; Department of Biological Sciences, Xi’an Jiaotong-Liverpool University, Suzhou, 215123, China; Department of Biological Sciences, Xi’an Jiaotong-Liverpool University, Suzhou, 215123, China; Institute of Systems, Molecular and Integrative Biology, University of Liverpool, Liverpool L7 8TX, UK; Department of Biological Sciences, Xi’an Jiaotong-Liverpool University, Suzhou, 215123, China; Department of Computer Science, University of Liverpool, Liverpool L7 8TX, UK; Department of Mathematical Sciences, Xi’an Jiaotong-Liverpool University, Suzhou, 215123, China; Department of Computer Science, University of Liverpool, Liverpool L7 8TX, UK; Institute of Life Course and Medical Sciences, University of Liverpool, Liverpool L7 8TX, UK; Institute of Systems, Molecular and Integrative Biology, University of Liverpool, Liverpool L7 8TX, UK; Department of Biological Sciences, Xi’an Jiaotong-Liverpool University, Suzhou, 215123, China; Institute of Systems, Molecular and Integrative Biology, University of Liverpool, Liverpool L7 8TX, UK; AI University Research Centre, Xi’an Jiaotong-Liverpool University, Suzhou, 215123, China; Department of Mathematical Sciences, Xi’an Jiaotong-Liverpool University, Suzhou, 215123, China; Key Laboratory of Ministry of Education for Gastrointestinal Cancer, School of Basic Medical Sciences, Fujian Medical University, Fuzhou, 350004, China; Department of Biological Sciences, Xi’an Jiaotong-Liverpool University, Suzhou, 215123, China; Institute of Life Course and Medical Sciences, University of Liverpool, Liverpool L7 8TX, UK

## Abstract

Recent advances in epitranscriptomics have unveiled functional associations between RNA modifications (RMs) and multiple human diseases, but distinguishing the functional or disease-related single nucleotide variants (SNVs) from the majority of ‘silent’ variants remains a major challenge. We previously developed the RMDisease database for unveiling the association between genetic variants and RMs concerning human disease pathogenesis. In this work, we present RMDisease v2.0, an updated database with expanded coverage. Using deep learning models and from 873 819 experimentally validated RM sites, we identified a total of 1 366 252 RM-associated variants that may affect (add or remove an RM site) 16 different types of RNA modifications (m^6^A, m^5^C, m^1^A, m^5^U, Ψ, m^6^Am, m^7^G, A-to-I, ac^4^C, Am, Cm, Um, Gm, hm^5^C, D and f^5^C) in 20 organisms (human, mouse, rat, zebrafish, maize, fruit fly, yeast, fission yeast, Arabidopsis, rice, chicken, goat, sheep, pig, cow, rhesus monkey, tomato, chimpanzee, green monkey and SARS-CoV-2). Among them, 14 749 disease- and 2441 trait-associated genetic variants may function via the perturbation of epitranscriptomic markers. RMDisease v2.0 should serve as a useful resource for studying the genetic drivers of phenotypes that lie within the epitranscriptome layer circuitry, and is freely accessible at: www.rnamd.org/rmdisease2.

## INTRODUCTION

Advances in high-throughput sequencing have revealed millions of single nucleotide variants (SNVs) in genomes. A key challenge lies in the functional annotation of genetic variants, especially if the mutations are synonymous or from the non-coding regions. There is increasing evidence that synonymous variants can affect essential biological functions via epigenetic regulation (1). Moreover, accurate identification of functional SNVs is crucial to better understand the molecular mechanisms underlying human diseases. To annotate the genetic variants with putative downstream mechanisms, enormous computational efforts have been made in exploring the effects of the mutations on various genomic phenomena, including post-transcriptional protein modification (2–8), transcriptional regulation (9,10), RNA–protein interaction (11), ceRNA networks (12), calpain cleavage (13), polyadenylation (14) and RNA modifications (15–18).

To date, >170 different types of post-transcriptional RNA modifications (RMs) have been detected, which occurs on different types of RNA and regulates nearly every stage of RNA life. Emerging evidence has revealed that dysregulation of the modification status is involved in multiple human diseases including cancer contexts (19,20). RMs have also been shown to play an essential role in regulating the function of tRNA and mitochondrial RNA. Covalent modifications such as 2′-*O*-methylation (Nm) within eukaryotic and prokaryotic tRNAs can inhibit the innate immune responses (21–23). Mitochondrial RMs have been reported to shape metabolic plasticity in metastatic cancers (24), and the specific locations of 5-methylcytosine (m^5^C) and its derivative 5-formylcytosine (f^5^C) on mitochondrial transcriptome can be a potential therapeutic target for metastasis.

To understand the effects of genetic variants on RMs, we have built a database of RM-associated variants with an emphasis on their potential disease associations (17). In RMDisease, by integrating human genetic variants and 303 426 experimentally validated modified sites from eight types of RMs, a total of 202 307 human RM-associated variants were identified and each labeled with the association level (AL). Among them, >4000 disease-relevant variants were annotated, shedding light on the disease mechanisms potentially acting through altering the epitranscriptome layer.

To date, various techniques have been developed to profile RMs. Among these detection techniques, MeRIP-Seq (or m^6^A-Seq) occupies the majority of the market. MeRIP-Seq enables transcriptome-wide RM profiling with a resolution of ∼100 nt (25,26). Besides MeRIP-Seq, many techniques have been developed to detect RMs at base resolution, such as m6A-CLIP (27), m6ACE-Seq (28), Nm-Seq (29), m7G-Seq (30), Pseudo-Seq (31), m1A-Seq (32), RNA-BisSeq (33), FICC-Seq (15), miCLIP-Seq (34), f5C-Seq (35) and Rhp-Seq (36). MeRIP-Seq together with high-resolution techniques revealed the transcriptomic profiles of RM sites from multiple species. These detected RM sites can be a valuable resource for computational analysis to unveil the functions of epitranscriptomes in gene regulation and their implication in human diseases (37–39). In response, efforts have been made to identify the RM-associated variants in human and mouse transcriptomes, resulting in the construction of databases such as RMDisease (17) and RMVar (18) (please refer to Supplementary Table S1 for a brief comparison in the coverage of databases serving a similar purpose). As data from more modification types and species are now available, it is necessary to extend our previous analyses to them.

**Figure 1.**
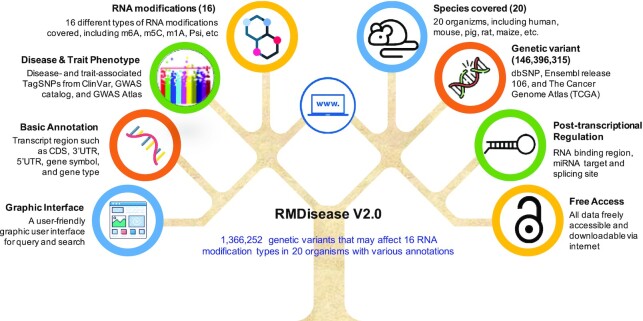
The overall design of RMDisease v2.0. RMDisease v2.0 was developed to decipher the effect of genetic factors on epitranscriptome disturbance. Currently, RMDisease v2.0 holds ∼1 360 000 RM-associated variants identified from 20 species, targeting 16 types of modification sites. In addition, ∼17 000 RM-associated variants are linked to specific disease or trait associations, with functional annotation of potentially involved post-transcriptional regulations, predicted RNA secondary structures and other informative resources. An enhanced web interface with real-time analysis functions is freely accessible at: www.rnamd.org/rmdisease2.

We have recently upgraded RMDisease to v2.0 by collecting all available RM-associated variants and annotating their potential involvement in diseases and traits. By integrating 873 819 experimentally validated modified sites and a total of 146 396 315 genetic variants, RMDisease v2.0 reports a total of 1 366 252 RM-associated variants that may affect (add or remove) modified sites of 16 types of RM [*N*^6^-methyladenosine (m^6^A), 5-methylcytosine (m^5^C), *N*^1^-methyladenosine (m^1^A), 5-methyluridine (m^5^U), pseudouridine (Ψ), *N*^6^,2′-*O*-dimethyladenosine (m^6^Am), *N*^7^-methylguanosine (m^7^G), adenosine to inosine (A-to-I), *N*^4^-acetylcytidine (ac^4^C), 2′-*O*-methylation (Am), 2′-*O*-methylation (Cm), 2′-*O*-methylation (Um), 2′-*O*-methylation (Gm), 5-hydroxymethylcytosine (hm^5^C), dihydrouridine (D) and 5-formylcytidine (f^5^C)] in 20 species (human, mouse, rat, zebrafish, maize, fruit fly, yeast, fission yeast, Arabidopsis, rice, chicken, goat, sheep, pig, cow, rhesus, tomato, chimpanzee, green monkey and SARS-CoV-2), including 14 749 disease- and 2441 trait-associated variants that may function through disturbing the epitranscriptome. In addition, RNA-binding protein (RBP)-binding sites, microRNA (miRNA) targets and splicing sites were annotated at each RM-associated variant to highlight potential effects on post-transcriptional regulation. The overall design of RMDisease v2.0 is outlined in Figure 1.

## MATERIALS AND METHODS

### Data resource

In RMDisease v2.0, we collected the epitranscriptome profiles of 16 types of RMs from 20 species, namely m^6^A (589 290 sites), m^5^C (150 412), m^1^A (32 758), m^5^U (3696), Ψ (7032), m^6^Am (2447), m^7^G (9951), A-to-I (52 760), ac^4^C (14 266), Am (1591), Cm (1878), Um (2253), Gm (1471), hm^5^C (1759), D (371) and f^5^C (1892), respectively. Specifically, the various types of RM sites were derived from 679 high-throughput sequencing samples by 21 sequencing techniques (please refer to Supplementary Tables S2 and S3). For RMs identified using base-resolution techniques, the genomic coordinates of modified residues were extracted from the corresponding GSE file or supplementary materials of their original publications. For the low-resolution MeRIP-Seq data, the raw FASTQ files were downloaded and re-processed using a common pipeline. Specifically, the raw reads were firstly aligned to the reference genome with hisat2, and the peak-calling process was then performed using exomePeak2 (40) with GC contents corrected.

The genetic variants analyzed in this study consist of two groups. The germline variants in various species were derived from dbSNP (v151) (41), Ensembl 2022 (Ensembl release 106) (42) and 1000 Genomes (Phase3 Mitochondrial Chromosome Variants set). The somatic variants were obtained from 27 different human cancer types in the Cancer Genome Atlas (TCGA) (release version v27.0-fix) (43). We considered in this study a total of 144 117 977 germline variants and 2 278 338 somatic variants, identified in 20 species, respectively (see Supplementary Table S4).

### Derivation of RNA modification-associated variants

An RM-associated variant is defined as the genetic mutation leading to either the gain or loss of a specific RM site, as predicted by our previously developed deep neural network model (44). The deep learning-based models were trained by modified residues from one modification type of a specific species. The tissue homogeneity assumption used is consistent with existing studies (17,18) while, in practice, little variation across training tissues is observed on the inference outcomes of cross-tissue validation (Supplementary Figure S1). The obtained RM-associated variants were further classified into three confidence levels. Specifically, these confidence levels were: (i) high: an experimentally validated RM site was directly altered by a genetic variant, resulting in the loss of its modified nucleotide; (ii) medium: a genetic variant altered a nucleotide within the 41 bp flanking window of a base-resolution modification site (the modified site lies in the center of the 41 bp sequence) or an experimentally validated RM-containing region (MeRIP-Seq with a resolution of ∼100 nt), leading to the loss of its modification status in the mutated sequence evaluated by the deep-learning model; and (iii) low: the transcriptome-wide prediction was performed for a genetic variant altering a nucleotide within the 41 bp flanking window centered on a modification site or the RM-containing region, resulting in the significant decrease or increase in the predicted probability of the modification status, compared between the original and the mutated sequence.

Using the same definition as RMDisease 1.0, we calculated the AL between genetic variant and RM site with the following:

(1) $$\begin{equation*}{\rm{AL}} = \left\{ {\begin{array}{@{}*{2}{c}@{}} {{\rm{2}}{P_{SNP}} - 2\max \left( {0.5,{P_{WT}}} \right)}&{{\rm{for\,\, gain}}}\\ {2{P_{WT}} - 2\max \left( {0.5,{P_{SNP}}} \right)}&{{\rm{for \,\,loss}}} \end{array}} \right.\end{equation*}$$

where *P*_*WT*_ and *P*_*SNP*_ represent the probability of RM status for the wild-type and mutated (SNP) sequences, respectively. The AL was then calculated. AL ranges from 0 to 1, with 1 indicating the greatest impact of the variant on the modification status. The statistical significance of AL was evaluated by calculating the *P*-values using the null distribution of AL from all genetic variants. We retained only the RM-associated variants passing a strict cut-off (AL >0.4 and *P*-value <0.05 or *P*-value <0.01 for species with an extremely low number of available variants) as predicted by the deep-learning model.

### Functional annotation of RNA modification-associated variants

To aid functional interpretation, we annotated the identified variants with genomic information such as transcript region [coding sequence (CDS), 3′-untranslated region (3′UTR), 5′UTR, start codon and stop codon], genome conservation [phastCons 100-way (45) and ConsRM score (46)], predicted RNA secondary structure information (47), mutation type (non-synonymous or synonymous variant), RS ID, TCGA barcode, gene annotation (Ensembl gene ID, gene symbol, gene type), and ANNOVAR package (48) for deleterious level predicted by SIFT (49), PolyPhen2 HVAR (50), PolyPhen2HDIV (50), LRT (51) and FATHMM (52). The tRNA information was annotated by extracting genomic ranges of tRNAs of seven species from GtRNAdb (53). RNAfold software (54) was used to calculate the secondary structure information of modification sites and to generate the corresponding graphic visualization. In addition, the potential post-transcriptional regulations of identified variants were annotated by checking whether they locate in RBP-binding regions from POSTAR2 (55), could be involved in miRNA–RNA interaction based on data from miRanda (56) and startBase2 (57), and/or lie at splicing sites as annotated by UCSC browser (58) annotation with a GT–AG role within 100 bp upstream and downstream of RM-associated variants.

### Disease and trait association analysis of RNA modification-associated variants

To explore potential epitranscriptome-related pathogenesis, an analysis was performed as follows. We integrated the human disease-associated variants and trait association TagSNPs from ClinVar (59), GWAS catalog (60), GWAS Atlas (61), Johnson and O’Donnell's database (62) and the National Genomics Data Center (63). The linkage disequilibrium (LD) analysis was computed for each trait association TagSNP using PLINK (64) software (parameters: –r2 –ld-snp-list –ld-window-kb 1000 –ld-window 10 –ld-window-r2 0.8). The RM-associated variants were then mapped to these disease-related variants, trait association TagSNPs and their LD mutations.

### Database and web interface implementation

RMDisease v2.0 web interfaces were constructed using HyperText Markup Language (HTML), Cascading Style Sheets (CSS) and Hypertext Preprocessor (PHP). All metadata was stored using MySQL tables. EChars was exploited to present statistical diagrams and the Jbrowse genome browser (65) was applied for interactive exploration and visualization of relevant genome coordinate-based records.

## RESULTS

### Database content

RMDisease v2.0 contains a total of 1 366 252 genetic variants that may affect (add or remove) various types of RMs in multiple species. This represents a 6-fold increase in RM-associated variants, as well as a significant expansion in covered species (from human only to 20 species) and type of RNA modification (from 8 to 16 types), compared with our previous version. Specifically, RMDisease v2.0 hosts RM-associated variants related to m^6^A (833 196), m^5^C (72 484), m^1^A (97 104), m^5^U (14 586), Ψ (84 950), m^6^Am (15 436), m^7^G (24 049), A-to-I (71 367), ac^4^C (45 891), Am (21 806), Cm (24 437), Um (37 313), Gm (19 623), hm^5^C (49), D (17) and f^5^C (3944), covering a variety of species in human (732 418), mouse (227 739), rat (1752), zebrafish (11 752), maize (1322), fruit fly (208), yeast (27 533), fission yeast (17), Arabidopsis (144 198), rice (10 438), chicken (14 679), goat (7860), sheep (18 439), pig (25 484), cow (64 275), rhesus (2442), tomato (64 830), chimpanzee (167), green monkey (137) and SARS-CoV-2 (10 562). Compared with another well-developed database (RMVar) focusing on the effect of genetic variants on RNA modifications (18), RMDisease v2.0 features a significant increase in both the types of RMs and the species supported (Supplementary Table S5). Eight new types of RMs (ac^4^C, hm^5^C, D, f^5^C, Am, Cm, Um and Gm) were covered for the first time, and the number of supported species was increased from two (human and mouse in RMVar) to 20, providing a more comprehensive landscape of the genetic factors potentially involved in epitranscriptome layer dysregulation. Please refer to Supplementary Tables S6–S8 for complete collections of RM-associated variants identified in RMDisease v2.0.

### Disease and trait association analysis

Next, the disease-related SNPs, trait-TagSNPs and their LD mutations were mapped to all RM-associated variants to unveil the phenotypes (disease or trait) potentially regulated at the epitranscriptome layer. We found that a total of 14 749 disease-relevant RM-associated variants are also linked to human diseases, which is more than three times larger than the previous version (Table 1). For example, for the m^6^A RM, 6477 genetic variants that may alter m^6^A status localized on 2026 genes were associated with 1709 known diseases and phenotypes according to the ClinVar and GWAS databases. In addition, 571 009 RM-associated somatic variants were also recorded in TCGA, linking them with 27 types of cancers (Supplementary Table S6). Furthermore, 2441 RM-associated variants were linked to various traits in four species (rice, sheep, cow and maize). We then calculated the disease and trait phenotypes that are most enriched within each type of RM; please refer to Supplementary Table S9 for a summary.

**Table 1.** Disease- and trait-associated RM-variants collected in RMDisease v2.0

		ClinVar			GWAS			GWAS Atlas		
Species	Modification type	SNP	Disease	Gene	SNP	Disease	Gene	SNP	Trait	Gene
Human	m^6^A	6,001	1,503	1,626	476	206	400	/	/	/
	m^1^A	976	446	473	80	57	73	/	/	/
	Ψ	827	307	268	90	58	78	/	/	/
	m^5^C	882	366	380	81	44	59	/	/	/
	m^5^U	402	111	59	44	21	25	/	/	/
	m^7^G	481	243	241	26	18	23	/	/	/
	m^6^Am	200	133	125	18	17	17	/	/	/
	A-to-I	866	457	436	75	49	69	/	/	/
	Am	517	180	134	19	16	18	/	/	/
	Cm	535	226	169	22	20	20	/	/	/
	Um	690	178	135	66	30	40	/	/	/
	Gm	471	189	135	30	26	29	/	/	/
	ac^4^C	798	388	340	76	60	74	/	/	/
Rice	m^6^A	/	/	/	/	/	/	757	98	89
Sheep	m^6^A	/	/	/	/	/	/	104	19	30
Cow	m^6^A	/	/	/	/	/	/	258	17	63
Maize	m^6^A	/	/	/	/	/	/	1,322	81	347

Please refer to Supplementary Tables S6–S8 for the complete collection in RMDisease v2.0.

### Enhanced web interface

We redesigned a user-friendly web interface to enables users to efficiently query, carry out customized searches and quickly download all collected RM-associated variants from the database. In addition, two new real-time data analysis modules were developed and presented on the web interface, namely VarFinder and Enrichment analysis tool. Users can upload a list of genetic variants in VCF format and perform inference on the database collections.

#### Query

**Figure 2.**
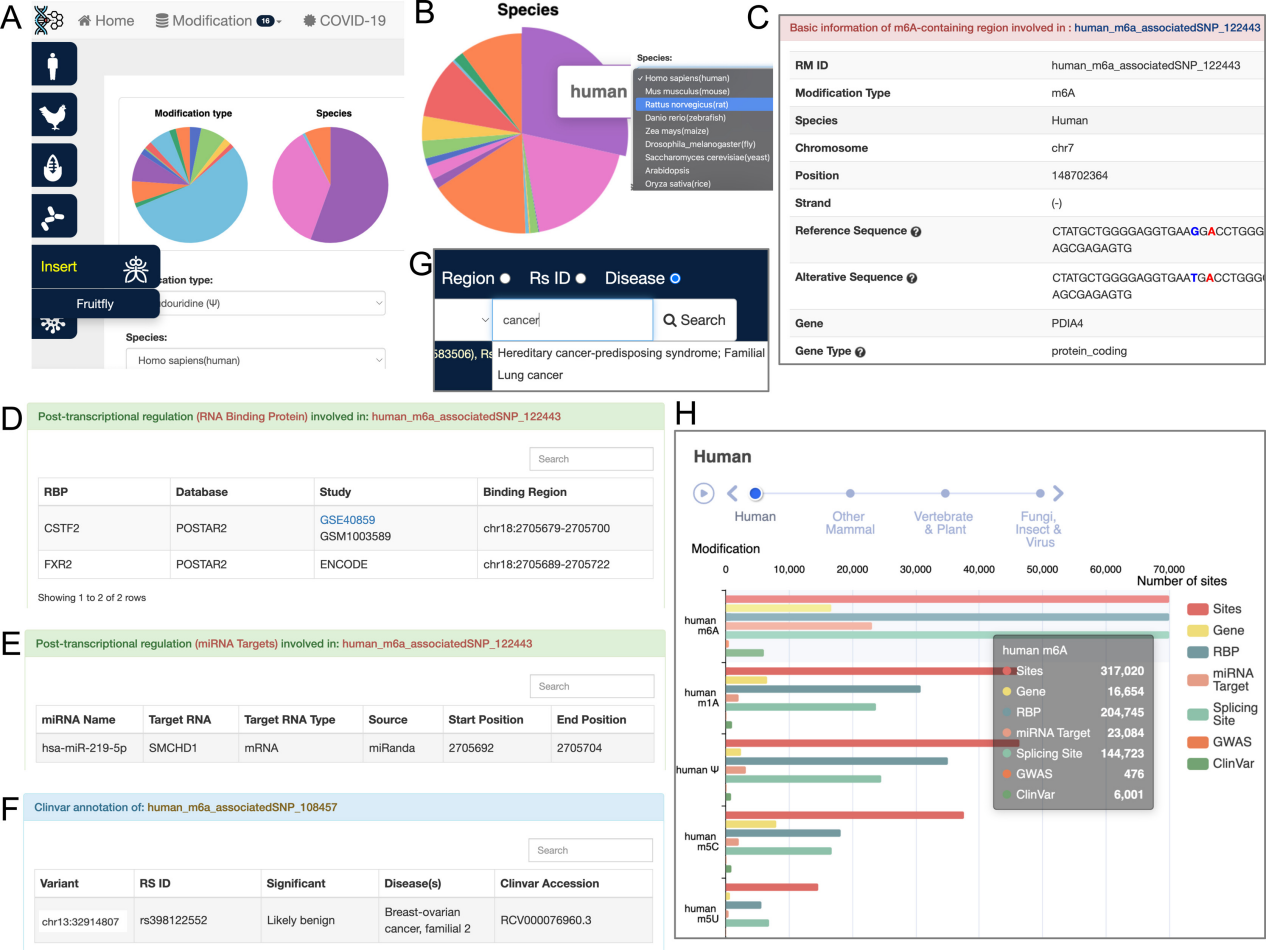
Contents of the RMDisease v2.0 database. (**A**) Users can query the RM-associated variants by species (left panel) or modification type (top panel). (**B**) A pie chart showing all available species under type of m^6^A RNA modification. (**C**) The detailed information of an RNA modification site. (**D, E**) The involved post-transcriptional regulations, including RBP-binding region and miRNA target. (**F**) The disease association of the RM-associated variant recorded in ClinVar. (**G**) Users can search related RM-associated variants using a specific disease phenotype. (**H**) An overview of all the data collected in RMDisease v2.0.

RMDisease v2.0 provides two modes to query all collected RM-associated variants. The user can query the database by selecting both species and the modification type (Figure 2A). For example, when users query the database by clicking the ‘N6-methyladenosine (m^6^A)’ button, the returned page will display m^6^A-associated variants identified with available species (Figure 2B). Users can further select a specific species and click the individual RM ID to check the summary table (Figure 2C), which includes basic information of the modification site and the associated variant(s), reference and mutated sequence, data source and post-transcriptional regulations involved (miRNA targeting, RBP-binding region and alternative splicing, Figure 2D–F). In addition, a statistics plot is provided for the visualization of the global data distribution (Figure 2H).

#### Search

In RMDisease v2.0, five kinds of search options are provided: Gene, Chromosome Region, RS ID, Disease Phenotypes and Trait Association. For example, searching by gene ‘FOXM1’ in human species in the search box of the RMDisease v2.0 front page returns 73 records from m^6^A modification, one record from m^1^A modification, 17 records from m^6^Am modification and 10 records from Gm modification. Users can further screen the results by adding multiple filters (e.g. modification type, gene type, conference level and functional annotation).

#### Disease and trait association

The disease and trait associations collected in RMDisease v2.0 can be exported in two ways. (i) Users can first query the database by their modification or species of interest, and then click the ‘GWAS’, ‘ClinVar’ or ‘Trait’ button from the corresponding filter columns. (ii) If users are interested in a specific disease or phenotype, keywords can be queried via the ‘Disease’ and ‘Trait’ options under the search box (Figure 2G). The query will return a full list of relevant data on a specific trait.

#### Download and share

All data collected in RMDisease v2.0 can be freely accessed and shared. Users can download all the data or their section of interest from the ‘Download’ page. Additionally, users can also access the application program interface (API) on the web interface, which offers a personalized query and download of all collected data. Please refer to the ‘Help’ and ‘API’ pages for more complete data descriptions.

#### Enhanced analysis tools

Two data analysis modules were firstly introduced. (i) Enrichment analysis: the statistical significance and fold enrichment of user-provided human variants over 33 TCGA cancer types and 16 modification types were calculated. Specifically, when users provide a list of human variants, the enrichment analysis calculates whether this variant set is significantly correlated to any specific type of TCGA cancer variants or modification disturbance, based on the statistical significance of the enrichment reported by the *P*-value of a binomial test. (ii) VarFinder: calculating the associations between a list of user-uploaded variants and 16 types of RNA modifications. Users can upload a list of interested variants to evaluate their potential effects over sites of a specific RM.

### Case study on COVID-19: spike glycoprotein

**Figure 3.**
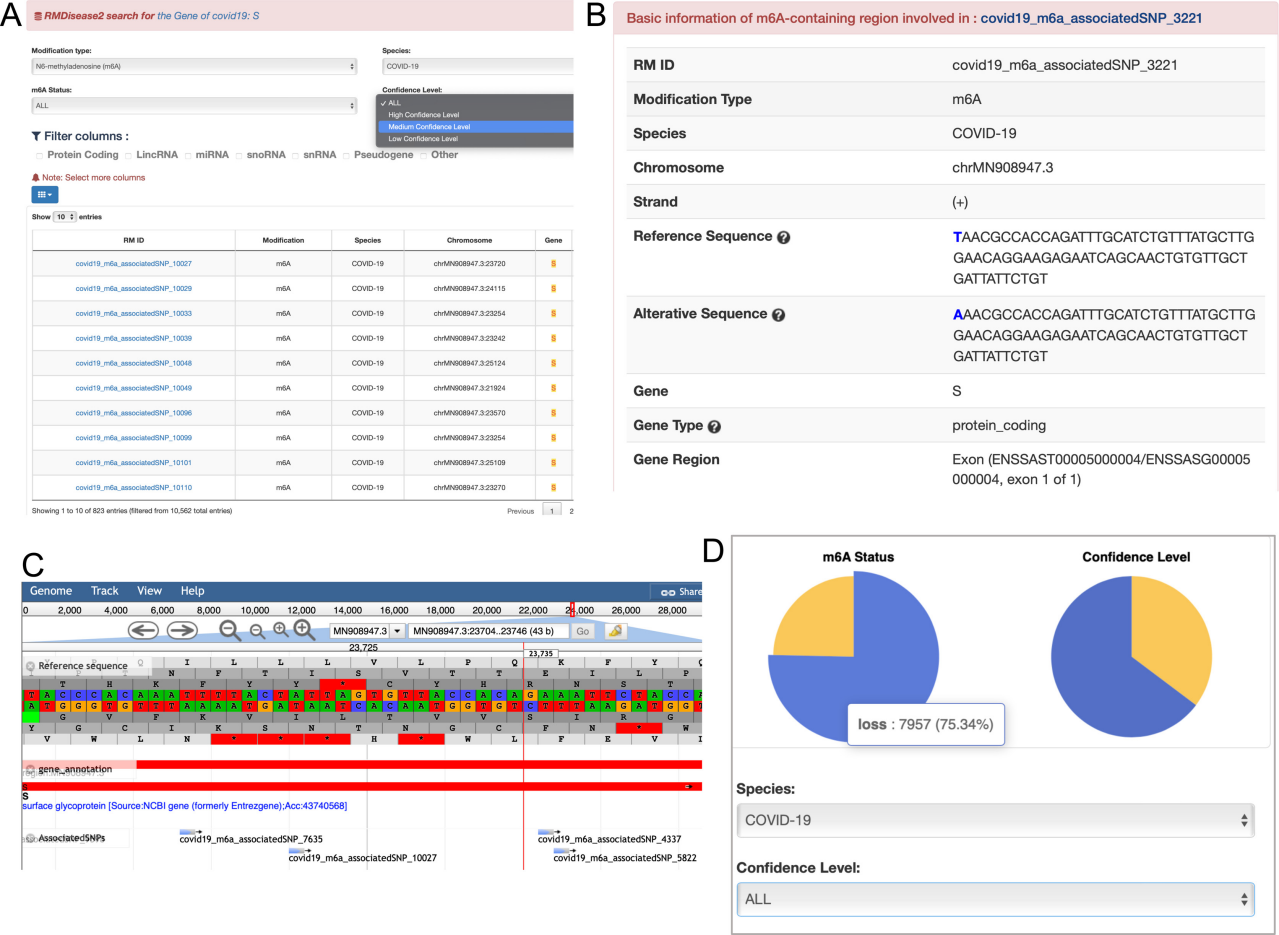
m^6^A-associated variants in the COVID-19 spike gene. (**A**) The search function enables users to search genetic variants located on a specific gene. (**B**) The basic information of a COVID19 m^6^A site destroyed by a genetic variant. (**C**) The Jbrowse genome browser offers to view the genome coordinate of the region of interest. (**D**) Besides a specific search, users can view all collected COVID-19 variants, comprising 7957 m^6^A-loss variants and 2605 m^6^A-gain variants.

The ‘COVID-19’ page of RMDisease v2.0 collects the m^6^A-affected variants located on COVID-19. Studies have confirmed that mutations in the COVID-19 spike gene (S) play a crucial role in infectivity enhancement and immune escape (66,67). m^6^A modifications in COVID-19 have also been reported to be associated with the innate immune response of the host cell (68). Of interest here are the m^6^A-affected variants located on the COVID-19 spike gene (S). Searching by gene ‘S’ in COVID-19 in the search box returns a total of 823 records (Figure 3A), comprising 197 m^6^A-gain variants and 626 m^6^A-loss variants. It is possible to further filter the records by confidence level; the user can select the ‘medium’ level so that only variants that may destroy experimentally validated m^6^A sites identified on COVID-19 spike genes remain. More information can be viewed by clicking a specific RM ID (Figure 3B), including supported study, gene type, gene region, association level, alternative sequence and Jbrowse genome browser (Figure 3C). Besides the spike gene, the user can view all COVID-19 m^6^A-affected variants collected in RMDisease v2.0 (Figure 3D).

## DISCUSSION

Increasing numbers of studies have reported that RNA modifications regulate essential biological processes and are involved in the mechanisms of multiple diseases. To further elucidate the genetic basis of epitranscriptome regulation, RMDisease v2.0 collected a total of 1 366 252 RM-associated variants that can alter 16 types of RM in 20 species. A large number of disease-/trait-associated variants are identified to elucidate the potential impact on phenotype of perturbations at the epitranscriptome layer.

Compared with RMDisease V1.0 and RMVar, substantial improvements have been made in RMDisease v2.0 to cover wider RM types and more species. The number of RM-associated variants presented in RMDisease v2.0 is six times more than the previous release. As high-throughput epitranscriptome data become increasingly available, advanced RM profiling techniques are continually being developed to identify previously uncovered modification sites, along with transcriptome-wide detection of new types of modification from various species. RMDisease will be continuously updated and expanded in the future to serve as a useful resource for the research communities of RM and genetics.

## DATA AVAILABILITY

All data used in this study are already publicly available in the GEO database, National Genomics Data Center, The Cancer Genome Atlas (TCGA), dbSNP (v151), 1000 Genome and Ensembl 2022 (Ensembl release 106). The accession number and detailed description can be found in Supplementary Tables S2–S4. All the identified RM-associated variants collected in RMDisease v2.0 are freely accessible at: www.rnamd.org/rmdisease2.

## Supplementary Material

gkac750_Supplemental_Files
