# Supplementary material for: RMDisease V2.0: an updated database of genetic variants that affect RNA modifications with disease and trait implication
Source: Nucleic Acids Res. 2022 Sep 5;51(D1):D1388–96. doi: 10.1093/nar/gkac750 (PMC9825452; doi:10.1093/nar/gkac750)
Supplement: gkac750_Supplemental_Files [file gkac750_supplemental_files.zip › Supplementary information.docx]

**Table S1 Comparing databases of genetic variants affecting RNA modifications**

|  | RMDisease  (v2.0) | RMDisease  (v1.0) [1] | RMVar [2] | m7GHub  [3] | m6AVar  [4] |
| --- | --- | --- | --- | --- | --- |
| Number of modification types | 16 | 8 | 9 | 1 | 1 |
| Number of species | 20 | 1 | 2 | 1 | 2 |
| Number of species with trait-associated RM-variant | 4 | - | - | - | - |
| Number of modifications with  disease-associated RM-variant | 13 | 8 | 9 | 1 | 1 |

**Note**: more detailed coverage of RMDisease, please refer to **Supplementary Table S5.**

**Table S5 Coverage of RMDisease V2.0**

| **Species** | **RNA Modification** | | | | | | | | | | | | | | | |
| --- | --- | --- | --- | --- | --- | --- | --- | --- | --- | --- | --- | --- | --- | --- | --- | --- |
|  | m^6^A | m^1^A | Psi | m^5^C | m^5^U | m^7^G | m^6^Am | I | ac^4^C | Am | Cm | Um | Gm | hm^5^C | D | f^5^C |
| Human | ✓ | ✓ | ✓ | ✓ | ✓ | ✓ | ✓ | ✓ | ✓ | ✓ | ✓ | ✓ | ✓ |  |  |  |
| Mouse | ✓ | ✓ | ✓ | ✓ |  |  |  | ✓ |  |  |  |  |  |  |  |  |
| Rat | ✓ |  |  |  |  |  |  |  |  |  |  |  |  |  |  |  |
| Zebrafish | ✓ |  |  | ✓ |  |  |  |  |  |  |  |  |  |  |  |  |
| Maize | ✓ |  |  |  |  |  |  |  |  |  |  |  |  |  |  |  |
| Fruit fly | ✓ |  |  |  |  |  |  |  |  |  |  |  |  | ✓ |  |  |
| Yeast | ✓ | ✓ | ✓ | ✓ |  |  |  |  |  |  |  |  |  |  |  | ✓ |
| Fission  yeast |  |  |  |  |  |  |  |  |  |  |  |  |  |  | ✓ |  |
| Arabidopsis | ✓ | ✓ | ✓ |  |  |  |  |  |  |  |  |  |  |  |  |  |
| Rice | ✓ |  |  |  |  |  |  |  |  |  |  |  |  |  |  |  |
| Chicken | ✓ |  |  |  |  |  |  |  |  |  |  |  |  |  |  |  |
| Goat | ✓ |  |  |  |  |  |  |  |  |  |  |  |  |  |  |  |
| Sheep | ✓ |  |  |  |  |  |  |  |  |  |  |  |  |  |  |  |
| Pig | ✓ |  |  |  |  |  |  |  |  |  |  |  |  |  |  |  |
| Cow | ✓ |  |  |  |  |  |  |  |  |  |  |  |  |  |  |  |
| Rhesus | ✓ |  |  |  |  |  |  |  |  |  |  |  |  |  |  |  |
| Tomato | ✓ |  |  |  |  |  |  |  |  |  |  |  |  |  |  |  |
| Green  monkey | ✓ |  |  |  |  |  |  |  |  |  |  |  |  |  |  |  |
| Chimpanzee | ✓ |  |  |  |  |  |  |  |  |  |  |  |  |  |  |  |
| COVID-19 | ✓ |  |  |  |  |  |  |  |  |  |  |  |  |  |  |  |

**Note:** Since epitranscriptome data is still scarce, only m^6^A, m^1^A, Psi and m^5^C-assocated genetic variants were identified in organisms other than human and mouse.

**Table S6 RM-associated variants collected in RMDisease v2.0**

(Part 1: human)

| **Modification**  **type** | **Conference**  **level** | **Germline variant** | | | **Somatic variant** | | | **Total** | | |
| --- | --- | --- | --- | --- | --- | --- | --- | --- | --- | --- |
|  |  | **Loss** | **Gain** | **Total** | **Loss** | **Gain** | **Total** | **Loss** | **Gain** | **Total** |
| m^6^A | High | 2,158 | **-** | 2,158 | 6,253 | **-** | 6,253 | 8,411 | **-** | 8,411 |
|  | Medium | 29,418 | **-** | 29,418 | 113,082 | **-** | 113,082 | 142,500 | **-** | 142,500 |
|  | Low | 13,113 | 21,774 | 34,887 | 54,108 | 82,950 | 137,058 | 67,221 | 104,724 | 171,945 |
| m^1^A | High | 300 | **-** | 300 | 176 | **-** | 176 | 476 | **-** | 476 |
|  | Medium | 6,016 | **-** | 6,016 | 12,430 | **-** | 12,430 | 18,446 | **-** | 18,446 |
|  | Low | 3,932 | 3,890 | 7,822 | 14,800 | 8,265 | 23,065 | 18,732 | 12,155 | 30,887 |
| Ψ | High | 11 | **-** | 11 | 31 | **-** | 31 | 42 | **-** | 42 |
|  | Medium | 564 | **-** | 564 | 1,782 | **-** | 1,782 | 2,346 | **-** | 2,346 |
|  | Low | 5,314 | 6,510 | 11,824 | 12,202 | 21,485 | 33,687 | 17,516 | 27,995 | 45,511 |
| m^5^C | High | 1,732 | **-** | 1,732 | 4,384 | **-** | 4,384 | 6,116 | **-** | 6,116 |
|  | Medium | 5,367 | **-** | 5,367 | 19,123 | **-** | 19,123 | 24,490 | **-** | 24,490 |
|  | Low | 4,133 | 871 | 5,004 | 1,867 | 339 | 2,206 | 6,000 | 1,210 | 7,210 |
| m^5^U | High | 29 | **-** | 29 | 7 | **-** | 7 | 36 | **-** | 36 |
|  | Medium | 272 | **-** | 272 | 235 | **-** | 235 | 507 | **-** | 507 |
|  | Low | 2,051 | 1,389 | 3,440 | 6,858 | 3,745 | 10,603 | 8,909 | 5,134 | 14,043 |
| m^7^G | High | 84 | **-** | 84 | 514 | **-** | 514 | 598 | **-** | 598 |
|  | Medium | 2,350 | **-** | 2,350 | 11,056 | **-** | 11,056 | 13,406 | **-** | 13,406 |
|  | Low | 660 | 1,426 | 2,086 | 2,281 | 5,678 | 7,959 | 2,941 | 7,104 | 10,045 |
| m^6^Am | High | 32 | **-** | 32 | 23 | **-** | 23 | 55 | **-** | 55 |
|  | Medium | 183 | **-** | 183 | 208 | **-** | 208 | 391 | **-** | 391 |
|  | Low | 1,937 | 1,440 | 3,377 | 7,893 | 3,720 | 11,613 | 9,830 | 5,160 | 14,990 |
| A-to-I | High | 111 | **-** | 111 | 112 | **-** | 112 | 223 | **-** | 223 |
|  | Medium | 4,006 | **-** | 4,006 | 11,252 | **-** | 11,252 | 15,258 | **-** | 15,258 |
|  | Low | 4,579 | 6,967 | 11,546 | 8,017 | 35,853 | 43,870 | 12,596 | 42,820 | 55,416 |
| Am | High | 1 | **-** | 1 | 14 | **-** | 14 | 15 | **-** | 15 |
|  | Medium | 72 | **-** | 72 | 552 | **-** | 552 | 624 | **-** | 624 |
|  | Low | 1,633 | 1,507 | 3,140 | 12,012 | 6,015 | 18,027 | 13,645 | 7,522 | 21,167 |
| Cm | High | 13 | **-** | 13 | 69 | **-** | 69 | 82 | **-** | 82 |
|  | Medium | 144 | **-** | 144 | 853 | **-** | 853 | 997 | **-** | 997 |
|  | Low | 1,547 | 2,382 | 3,929 | 9,025 | 10,404 | 19,429 | 10,572 | 12,786 | 23,358 |
| Um | High | 7 | **-** | 7 | 29 | **-** | 29 | 36 | **-** | 36 |
|  | Medium | 262 | **-** | 262 | 1,459 | **-** | 1,459 | 1,721 | **-** | 1,721 |
|  | Low | 3,417 | 3,404 | 6,821 | 18,130 | 10,605 | 28,735 | 21,547 | 14,009 | 35,556 |
| Gm | High | 2 | **-** | 2 | 75 | **-** | 75 | 77 | **-** | 77 |
|  | Medium | 72 | **-** | 72 | 353 | **-** | 353 | 425 | **-** | 425 |
|  | Low | 1,257 | 2,373 | 3,630 | 5,778 | 9,713 | 15,491 | 7,035 | 12,086 | 19,121 |
| ac^4^C | Medium | 9,627 | **-** | 9,627 | 30,247 | 0 | 30,247 | 39,874 | **-** | 39,874 |
|  | Low | 567 | 498 | 1,065 | 3,868 | 1,084 | 4,952 | 4,435 | 1,582 | 6,017 |

**Note:** The RM-associated variants classified into high confidence level refer to mutations directly destroying modified nucleotides, which requires modification sites available from base-resolution techniques.

**Table S7 RM-associated variants collected in RMDisease v2.0**

(Part 2: mouse, rat, zebra fish, fruit fly, yeast and Arabidopsis)

| **Species** | **Modification**  **type** | **Conference**  **level** | **Germline variant** | | | **Total** | | |
| --- | --- | --- | --- | --- | --- | --- | --- | --- |
|  |  |  | **Loss** | **Gain** | **Total** | **Loss** | **Gain** | **Total** |
| Mouse | m^6^A | High | 3,316 | - | 3,316 | 3,316 | - | 3,316 |
|  |  | Medium | 49,296 | - | 49,296 | 49,296 | - | 49,296 |
|  |  | Low | 34,202 | 36,136 | 70,338 | 34,202 | 36,136 | 70,338 |
|  | m^1^A | High | 165 | - | 165 | 165 | - | 165 |
|  |  | Medium | 995 | - | 995 | 995 | - | 995 |
|  |  | Low | 22,802 | 21,490 | 44,292 | 22,802 | 21,490 | 44,292 |
|  | m^5^C | High | 313 | - | 313 | 313 | - | 313 |
|  |  | Medium | 6,819 | - | 6,819 | 6,819 | - | 6,819 |
|  |  | Low | 13,302 | 8,078 | 21,380 | 13,302 | 8,078 | 21,380 |
|  | Ψ | High | 30 | - | 30 | 30 | - | 30 |
|  |  | Medium | 1,253 | - | 1,253 | 1,253 | - | 1,253 |
|  |  | Low | 13,564 | 15,508 | 29,072 | 13,564 | 15,508 | 29,072 |
|  | A-to-I | High | 29 | - | 29 | 29 | - | 29 |
|  |  | Medium | 42 | - | 42 | 42 | - | 42 |
|  |  | Low | 221 | 178 | 399 | 221 | 178 | 399 |
| Rat | m^6^A | High | 13 | - | 13 | 13 | - | 13 |
|  |  | Medium | 132 | - | 132 | 132 | - | 132 |
|  |  | Low | 786 | 821 | 1,607 | 786 | 821 | 1,607 |
| Zebrafish | m^6^A | High | 972 | - | 972 | 972 | - | 972 |
|  |  | Medium | 5,167 | - | 5,167 | 5,167 | - | 5,167 |
|  |  | Low | 1,181 | 1,193 | 2,374 | 1,181 | 1,193 | 2,374 |
|  | m^5^C | High | 77 | - | 77 | 77 | - | 77 |
|  |  | Medium | 2,436 | - | 2,436 | 2,436 | - | 2,436 |
|  |  | Low | 350 | 376 | 726 | 350 | 376 | 726 |
| Fruit fly | m^6^A | High | 0 | - | 0 | 0 | - | 0 |
|  |  | Medium | 10 | - | 10 | 10 | - | 10 |
|  |  | Low | 71 | 78 | 149 | 71 | 78 | 149 |
|  | hm^5^C | Medium | 5 | - | 5 | 5 | - | 5 |
|  |  | Low | 25 | 19 | 44 | 25 | 19 | 44 |
| Yeast | m^6^A | High | 462 | 0 | 462 | 462 | 0 | 462 |
|  |  | Medium | 2,227 | 0 | 2,227 | 2,227 | 0 | 2,227 |
|  |  | Low | 7,066 | 9,209 | 16,275 | 7,066 | 9,209 | 16,275 |
|  | m^1^A | High | 9 | - | 9 | 9 | - | 9 |
|  |  | Medium | 378 | - | 378 | 378 | - | 378 |
|  |  | Low | 734 | 722 | 1,456 | 734 | 722 | 1,456 |
|  | Ψ | High | 9 | - | 9 | 9 | - | 9 |
|  |  | Medium | 328 | - | 328 | 328 | - | 328 |
|  |  | Low | 126 | 175 | 301 | 126 | 175 | 301 |
|  | m^5^C | High | 30 | - | 30 | 30 | - | 30 |
|  |  | Medium | 942 | - | 942 | 942 | - | 942 |
|  |  | Low | 621 | 551 | 1,172 | 621 | 551 | 1,172 |
|  | f^5^C | High | 46 | - | 46 | 46 | - | 46 |
|  |  | Medium | 1,095 | - | 1,095 | 1,095 | - | 1,095 |
|  |  | Low | 569 | 2,234 | 2,803 | 569 | 2,234 | 2,803 |
| Arabidopsis | m^6^A | High | 5,295 | - | 5,295 | 5,295 | - | 5,295 |
|  |  | Medium | 30,970 | - | 30,970 | 30,970 | - | 30,970 |
|  |  | Low | 40,589 | 60,513 | 101,102 | 40,589 | 60,513 | 101,102 |
|  | Ψ | High | 16 | - | 16 | 16 | - | 16 |
|  |  | Medium | 1,003 | - | 1,003 | 1,003 | - | 1,003 |
|  |  | Low | 2,626 | 2,413 | 5,039 | 2,626 | 2,413 | 5,039 |
|  | m^5^C | High | 22 | - | 22 | 22 | - | 22 |
|  |  | Medium | 732 | - | 732 | 732 | - | 732 |
|  |  | Low | 11 | 8 | 19 | 11 | 8 | 19 |

**Note:** The RM-associated variants classified into high confidence level refer to mutations directly destroying modified nucleotides, which requires modification sites available from base-resolution techniques.

**Table S8 RM-associated variants collected in RMDisease v2.0**

(Part 3: rice, chicken, goat, sheep, pig, cow, tomato, chimpanzee, rhesus, etc.)

| **Species** | **Modification**  **type** | **Conference**  **level** | **Germline variant** | | | **Total** | | |
| --- | --- | --- | --- | --- | --- | --- | --- | --- |
|  |  |  | **Loss** | **Gain** | **Total** | **Loss** | **Gain** | **Total** |
| S.pombe | D | High | 1 | - | 1 | 1 | - | 1 |
|  |  | Medium | 7 | - | 7 | 7 | - | 7 |
|  |  | Low | 7 | 2 | 9 | 7 | 2 | 9 |
| Rice | m^6^A | Medium | 8,634 | - | 8,634 | 8,634 | - | 8,634 |
|  |  | Low | 866 | 938 | 1,804 | 866 | 938 | 1,804 |
| Chicken | m^6^A | Medium | 9,024 | - | 9,024 | 9,024 | - | 9,024 |
|  |  | Low | 2,974 | 2,681 | 5,655 | 2,974 | 2,681 | 5,655 |
| Goat | m^6^A | Medium | 6,941 | - | 6,941 | 6,941 | - | 6,941 |
|  |  | Low | 571 | 348 | 919 | 571 | 348 | 919 |
| Sheep | m^6^A | Medium | 16,974 | - | 16,974 | 16,974 | - | 16,974 |
|  |  | Low | 640 | 825 | 1,465 | 640 | 825 | 1,465 |
| Pig | m^6^A | Medium | 11,454 | - | 11,454 | 11,454 | - | 11,454 |
|  |  | Low | 6,776 | 7,254 | 14,030 | 6,776 | 7,254 | 14,030 |
| Cow | m^6^A | Medium | 38,463 | - | 38,463 | 38,463 | - | 38,463 |
|  |  | Low | 13,389 | 12,423 | 25,812 | 13,389 | 12,423 | 25,812 |
| Tomato | m^6^A | Medium | 50,007 | - | 50,007 | 50,007 | - | 50,007 |
|  |  | Low | 8,265 | 6,558 | 14,823 | 8,265 | 6,558 | 14,823 |
| Chimpanzee | m^6^A | Medium | 109 | - | 109 | 109 | - | 109 |
|  |  | Low | 46 | 12 | 58 | 46 | 12 | 58 |
| Rhesus | m^6^A | Medium | 2,093 | - | 2,093 | 2,093 | - | 2,093 |
|  |  | Low | 195 | 154 | 349 | 195 | 154 | 349 |
| COVID-19 | m^6^A | Medium | 3,723 | - | 3,723 | 3,723 | - | 3,723 |
|  |  | Low | 4,234 | 2,605 | 6,839 | 4,234 | 2,605 | 6,839 |
| Green  Monkey | m^6^A | Medium | 2 | - | 2 | 2 | - | 2 |
|  |  | Low | 82 | 53 | 135 | 82 | 53 | 135 |
| Maize | m^6^A | Medium | 508 | - | 508 | 508 | - | 508 |
|  |  | Low | 142 | 672 | 814 | 142 | 672 | 814 |

**Note:** The RM-associated variants classified into high confidence level refer to mutations directly destroying modified nucleotides, which requires modification sites available from base-resolution techniques.

**Table S9 Diseases and trait with the most RM-associated variants**

| Species | Disease/trait  name | #Variant | Study  accession | RM  Type | Source |
| --- | --- | --- | --- | --- | --- |
| Human | Hereditary cancer-predisposing syndrome | 233 | RCV000131276.2 | m^6^A | ClinVar |
|  | Charcot-Marie-Tooth, Type 4 | 19 | RCV000322591.1 | m^5^U |  |
|  | Mitochondrial complex II deficiency | 19 | RCV000232220.2 | m^7^G |  |
|  | Hereditary cancer-predisposing syndrome | 39 | RCV000131276.2 | m^1^A |  |
|  | Adenocarcinoma of lung | 6 | RCV000444227.1 | m^6^Am |  |
|  | Hereditary cancer-predisposing syndrome | 24 | RCV000131276.2 | Ψ |  |
|  | Breast-ovarian cancer, familial 1 | 16 | RCV000211047.1 | A-to-I |  |
|  | Hereditary cancer-predisposing syndrome | 11 | RCV000131276.2 | m^5^C |  |
|  | Limb-girdle muscular dystrophy, type 2J | 55 | RCV000457077.1 | Am |  |
|  | Microcephalic Osteodysplastic Primordial Dwarfism | 13 | RCV000359776.1 | Cm |  |
|  | Emery-Dreifuss muscular dystrophy | 28 | RCV000403709.1 | Um |  |
|  | Cardiomyopathy, ARVC | 17 | RCV000380049.1 | Gm |  |
|  | Collagen VI-related myopathy | 21 | RCV000384340.1 | ac^4^C |  |
| Rice | Germination index | 70 | PPTO:0000610 | m^6^A | GWAS Atlas |
| Sheep | Teat number | 26 | APTO:0000112 |  |  |
| Cow | Milking speed | 156 | APTO:0000157 |  |  |
| Maize | Plant height | 190 | PPTO:0000126 |  |  |


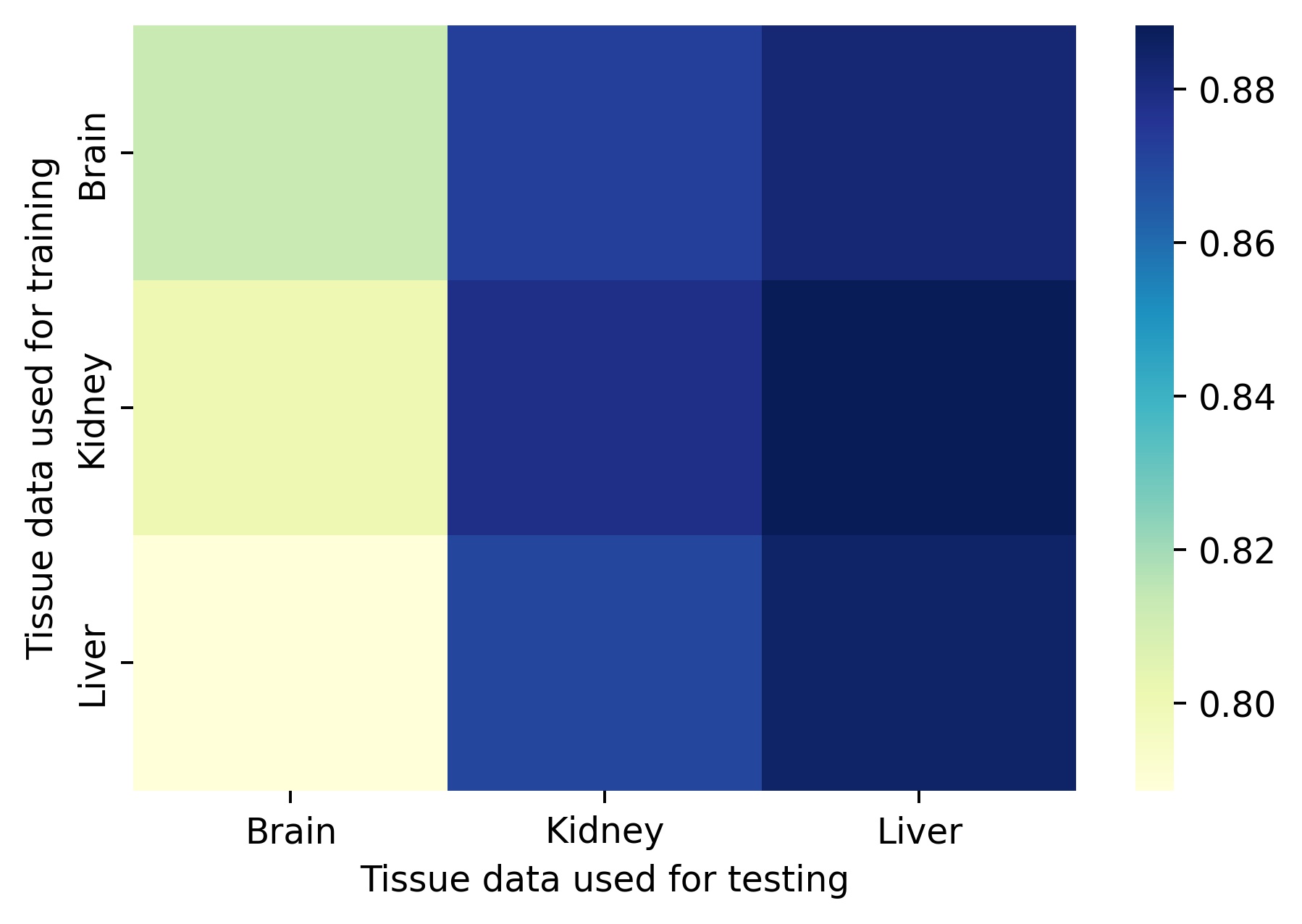


**Figure S1.** Cross-tissue validation. For each specific testing tissue, the model trained on the other tissue performed almost identically to the model trained on the same tissue, suggesting that the model only learned the general sequential pattern of m6A rather than the tissue-specific context.

**REFERENCE**

1. Chen, K., et al., *RMDisease: a database of genetic variants that affect RNA modifications, with implications for epitranscriptome pathogenesis.* Nucleic Acids Res, 2021. **49**(D1): p. D1396-D1404.

2. Luo, X., et al., *RMVar: an updated database of functional variants involved in RNA modifications.* Nucleic Acids Res, 2021. **49**(D1): p. D1405-D1412.

3. Song, B., et al., *m7GHub: deciphering the location, regulation and pathogenesis of internal mRNA N7-methylguanosine (m7G) sites in human.* Bioinformatics, 2020. **36**(11): p. 3528-3536.

4. Zheng, Y., et al., *m6AVar: a database of functional variants involved in m6A modification.* Nucleic Acids Res, 2018. **46**(D1): p. D139-D145.
